# Towards a carbon-negative sustainable bio-based economy

**DOI:** 10.3389/fpls.2013.00174

**Published:** 2013-06-03

**Authors:** Bartel Vanholme, Tom Desmet, Frederik Ronsse, Korneel Rabaey, Frank Van Breusegem, Marjan De Mey, Wim Soetaert, Wout Boerjan

**Affiliations:** ^1^Department of Plant Systems Biology, Flanders Institute for BiotechnologyGent, Belgium; ^2^Department of Plant Biotechnology and Bioinformatics, Ghent UniversityGent, Belgium; ^3^Department of Biochemical and Microbial Technology, Centre of Expertise – Industrial Biotechnology and Biocatalysis, Ghent UniversityGent, Belgium; ^4^Department of Biosystems Engineering, Ghent UniversityGent, Belgium; ^5^Laboratory of Microbial Ecology and Technology, Ghent UniversityGent, Belgium; ^6^Centre for Microbial Electrosynthesis, The University of QueenslandBrisbane, Australia; ^7^Advanced Water Management Centre, The University of QueenslandBrisbane, Australia

**Keywords:** biomass, lignocellulose, saccharification, fermentation, anaerobic digestion, pyrolysis, biochar

## Abstract

The bio-based economy relies on sustainable, plant-derived resources for fuels, chemicals, materials, food and feed rather than on the evanescent usage of fossil resources. The cornerstone of this economy is the biorefinery, in which renewable resources are intelligently converted to a plethora of products, maximizing the valorization of the feedstocks. Innovation is a prerequisite to move a fossil-based economy toward sustainable alternatives, and the viability of the bio-based economy depends on the integration between plant (green) and industrial (white) biotechnology. Green biotechnology deals with primary production through the improvement of biomass crops, while white biotechnology deals with the conversion of biomass into products and energy. Waste streams are minimized during these processes or partly converted to biogas, which can be used to power the processing pipeline. The sustainability of this economy is guaranteed by a third technology pillar that uses thermochemical conversion to valorize waste streams and fix residual carbon as biochar in the soil, hence creating a carbon-negative cycle. These three different multidisciplinary pillars interact through the value chain of the bio-based economy.

## THE BIO-BASED ECONOMY

With a rapidly growing world population and increasing wealth, mainly due to rising incomes in developing countries, we are facing the challenge to provide our economy with sufficient energy to guarantee our modern standard of living. Currently, the world’s economy is running on hydrocarbons that were formed over millions of years by decomposing plant and animal remains (Hook et al., 2010). These finite resources are being consumed at an increasing speed in an unsustainable way and the resulting release of carbon dioxide into the atmosphere is considered one of the key factors causing current global climate changes. In addition, fossil fuel reserves are at the base of international political and economic conflicts, mainly because these resources are unequally distributed over the globe (O’Lear, 2004). These different concerns have led to an increasing focus on alternative renewable and sustainable resources such as solar, wind, geothermal, hydroelectric, and wave energy. Although none of the developed technologies can solely fulfill our current energy needs, an integrated approach combined with a general concern for energy saving and improved energy-efficient technologies can definitely shift our conventional way of living toward sustainability (Lund, 2007).

**FIGURE 1:**
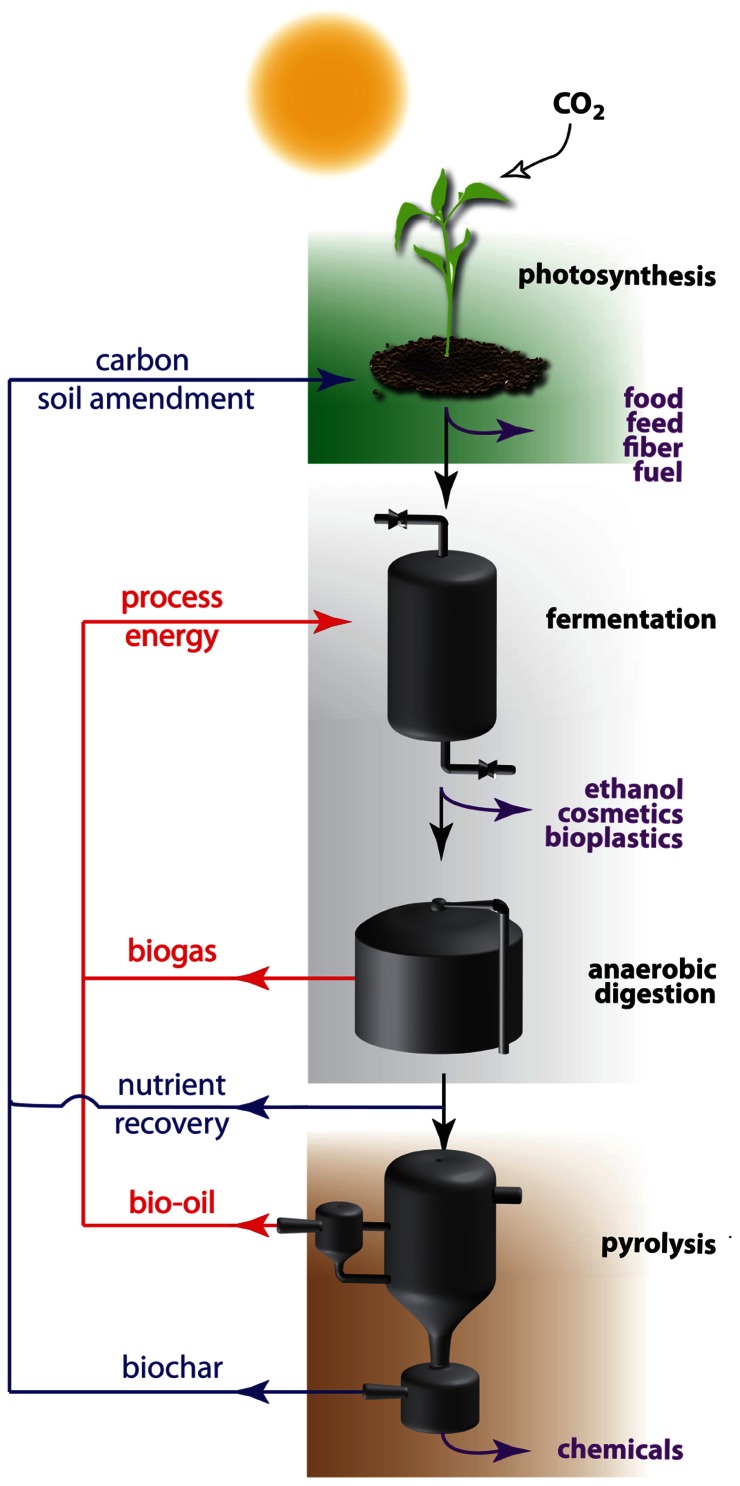
**Recycling of energy and nutrients within the carbon-negative bio-based economy**. The carbon-negative bio-based economy is based on three integrated processes: green biotechnology (top), white biotechnology (middle), and thermochemical conversion (bottom). Plants use solar energy to convert carbon dioxide into biomass, mainly plant cell walls with cellulose as most abundant polymer. This polymer is enzymatically converted to glucose monomers (saccharification) which are used as carbon source by microorganisms to produce chemical compounds, among which bioethanol. The efficiency of this process is mainly dependent on the recalcitrance of the cell wall that is considerably reduced by physical, thermal, or chemical pretreatments of the biomass. Waste streams are minimized or concentrated to feed anaerobic digesters for the production of biogas that can be integrated in the system. Rest fractions are converted into added value compounds, energy, or biochar by pyrolysis. The latter is used as a long acting soil amendment on the field. During passage over the different segments, specific fractions of the initial biomass can leave the processing treadmill for valorization into bio-based products (purple arrows), while carbon and nutrient waste streams are recycled as soil additive (blue arrows), or as energy (red arrows).

In a sustainable economy, not only energy, but also materials will be derived from renewable resources. Here, plant biomass has an important advantage over the mentioned energy resources as it is an easy accessible source for both energy and materials (**Figure 1**). Biomass is produced by photosynthesis, a biochemical process that uses solar energy to convert atmospheric carbon dioxide into carbohydrates. Initial attempts were made to launch a bio-based economy running on vegetable oils and on sugars derived from starch or sucrose. These so-called easily accessible first generation feedstocks are in direct competition with the food chain, and hence are not without controversy (Williams, 2008). As an alternative, feedstocks of the second generation were introduced. These refer to the entire plant biomass, which is mainly composed of plant cell wall polysaccharides. The annual amount of biomass produced by land plants is enormous and estimated numbers vary between 10 and 200 × 10^9^ tons (Pauly and Keegstra, 2008; Chandel et al., 2010; Kalluri and Keller, 2010). Roughly 70% of this biomass is made up of plant cell walls of which approximately three quarters are polysaccharides. Cellulose is a major polysaccharide of the cell wall, and this linear polymer of β-1,4-linked glucose units is considered to be the world’s most abundant biopolymer (Pauly and Keegstra, 2008). Glucose is an ideal carbon source to feed the bio-based economy, since it is easily converted by microorganisms and enzymes into ethanol and a variety of chemical compounds. In a sustainable production process, the remaining biomass is subsequently concentrated and processed to biogas by anaerobic digestion after which the residual waste fractions are converted into bio-oil or biochar by pyrolysis (i.e., heating under an oxygen-limited environment; Lehmann, 2007; Lehmann and Joseph, 2009). The initial biomass, as well as the derived biogas, bio-oil and fermentation products can all be used as energy source. The biochar produced at the end of the processing pipeline is a carbon-rich, recalcitrant product that contains all residual nutrients. Returning it to agricultural land closes the nutrient loop and sequesters atmospheric carbon, potentially creating a sustainable and carbon-negative cycle (Mathews, 2008).

The transition from the current fossil fuel-based economy toward a biomass-based economy requires the interplay of a wide variety of technologies. As the bio-based economy is established on two fundamental biological processes, being photosynthesis and fermentation, biotechnology is quickly becoming a technological cornerstone for the further development of a sustainable economy and plays a key role in the bio-economy value chain. Plant (green) biotechnology is important for the primary production of optimized biomass through the improvement of crops, whereas industrial (white) biotechnology is involved in improving the conversion efficiency of these renewable resources into a wide range of products. The third technology pillar supporting the carbon-negative bio-based economy focusses on the conversion of generated waste streams to added value products, being bio-oil and biochar. Each of the pillars needs to be optimized and the cross-disciplinary fields integrated to achieve a viable and sustainable alternative for our current economy based on fossil fuels (**Figure 1**).

## THE GREEN BIOTECHNOLOGY PILLAR: PRIMARY BIOMASS PRODUCTION

### INCREASING YIELD AND REDUCING STRESS

To develop a carbon-negative and sustainable bio-based economy, it is important to maximize the biomass yield per unit land. This obviously starts with the selection of the most appropriate biomass crops, which depends largely on the geographical location of the field. Ideally, these crops are able to produce substantial amounts of biomass over a short period of time, with minimal requirements for nutrient and water input. From this perspective, perennial plants like *Miscanthus*, poplar and willow are generally considered the preferred choice (Sanderson and Adler, 2008). A single planting effort, followed by multiple harvests in the subsequent years, combined with decreased needs for nutrient supply and pest control strategies compared to annual plants, results in a higher net energy balance. In addition, their longer roots offer significant soil protection effects, thereby reducing nutrient losses and rendering plants more tolerant against severe drought conditions. Plantations on marginal and waste lands avoid competition for arable land that is more suited for food and feed crops. Interestingly, a recent study discussed the potential of wild perennial herbaceous vegetation as an alternative for the intentionally grown crops on marginal lands (Gelfand et al., 2013). Although the concept of exploiting marginal land for biomass production is attractive, the high investment costs combined with the relatively low return are major bottlenecks that withhold the further exploitation of these areas. To reduce the costs related to transport and logistics, biomass processing could be performed locally or the process could be implemented into existing agricultural infrastructure (Dweikat et al., 2012).

Besides selecting the most appropriate crops, there is considerable potential in optimizing the plants’ physiological processes toward increased biomass production. The primary and key process in biomass production is photosynthesis, as it converts solar energy into sugars. This process is surprisingly inefficient (Larkum, 2010), since only a minor fraction of the absorbed solar energy is effectively incorporated into the biomass [around 1% compared to the 15–25% energy conversion efficiency of current photovoltaic cells (Green et al., 2013)]. Improving this energy conversion efficiency is a promising although challenging approach to increase yield and can be tackled at different levels: e.g., modifications of the plant architecture (Stamm et al., 2012) or leaf canopy to maximize the capture of sunlight (Zhu et al., 2012); increasing the stomatal density to improve the photosynthetic capacity (Tanaka et al., 2013); engineering C3 plants with a more efficient C4 metabolism and physiology (Zhu et al., 2010; Langdale, 2011; Covshoff and Hibberd, 2012); or improving enzymes catalyzing rate limiting steps in carbon fixation. The most important and best studied target for the latter approach is the key enzyme Rubisco (ribulose-1,5-biphosphate carboxylase/oxygenase), catalyzing the first step in the fixation of atmospheric carbon dioxide. Besides being slow, the enzyme lacks specificity, frequently incorporating oxygen rather than carbon dioxide. Due to the overall importance of this enzyme, even the slightest improvement in performance should have a significant effect on biomass production (Parry et al., 2013). However, progress in this field is rather slow (Blankenship et al., 2011) and other targeted approaches such as pathway shuffling to reduce the loss of carbon when oxygen is incorporated (Kebeish et al., 2007; Peterhansel et al., 2013), or improving photosynthetic electron flow in the thylakoid membrane, turned out to be more successful (Chida et al., 2007).

To find new potential candidate genes or pathways involved in yield improvement, integrated and system-wide approaches are being applied. These require computational tools to construct molecular models, describing growth regulation in an integrated way. Over the years this has resulted in the identification of a plethora of genes linked to increased biomass production. These “intrinsic yield genes” (IYGs) are involved in various processes and the functional study of these candidate genes is an ongoing process. Although their interrelationship is largely unknown, transgenic plants that overexpress/downregulate more than one IYG frequently reveal additive or synergistic effects on plant yield (Gonzalez et al., 2009), indicating that a combination of genes may have more potential to increase biomass in target crops than engineering the expression of individual genes (De Veylder et al., 2007). Also varieties that are able to cope with fluctuating and adverse environmental conditions, like drought, temperature, and salt stress, are needed, especially when considering that energy crops will be cultivated on marginal lands. When plants are exposed to abiotic stress, gene expression is altered to induce protective effects. These involve a complex regulatory network that mediates morphological, physiological, biochemical, and molecular changes. A better understanding of these events will be key in breeding plants with increased resistance to abiotic stress. Breeding crop varieties with improved performance during suboptimal growth conditions is currently one of the ambitious, but crucial objectives of plant biotechnology. In the model plant *Arabidopsis*, the function of at least 150 genes was correlated with increased stress tolerance. These so-called “stress tolerance genes” (STG) can be functionally categorized in two broad groups: executors of stress tolerance (protective enzymes, detoxifying proteins, ion transporters, antioxidants) and signal transducers (kinases, phosphatases, transcription factors…; Hirayama and Shinozaki, 2010; Skirycz et al., 2011).

Once analyzed in *Arabidopsis*, interesting findings have to be translated to economically valuable crops (Vinocur and Altman, 2005; Yamaguchi and Blumwald, 2005; Chew and Halliday, 2011). Successful examples of such transition were the overexpression of an *Arabidopsis* transcription factor and of bacterial RNA chaperones in maize leading to improved performance on water limited fields (Nelson et al., 2007; Castiglioni et al., 2008). Success of translational research is not guaranteed and depends to some extend on the genetic distance between *Arabidopsis* and the crop of interest. To reduce this distance, new species have been introduced such as the weedy *Setaria viridis* and domesticated *S. italica*, which are considered models for Panicoid grasses (including maize, sugarcane, and *Miscanthus*; Brutnell et al., 2010; Li and Brutnell, 2011). Another obstacle encumbering translational research is the genetic complexity of many crops which asks for new bioinformatics tools that can deal with high ploidy levels, allelic variation, low gene coverage, and highly repetitive sequences (Dal-Bianco et al., 2012). Finally, environmental conditions affect the success rate of translational research. Most studies in *Arabidopsis* focus on short-term and harsh stress treatments that seldom reflect natural conditions in the field, where combinations and fluctuating periods of stress occur. From an agronomic point of view, it is more relevant to assess the stress effects on plant growth and yield with readouts that span the whole life-cycle of the crop (Skirycz et al., 2011). Consequently, field trials are essential to extrapolate the lab results and analyze the plant’s performance under suboptimal growth conditions that are closer to real life (Pilate et al., 2002). This is especially the case for biomass crops, since cell wall composition can differ considerably between field- and greenhouse-grown plants, making greenhouse-based screenings not always effective in selecting plants with improved field performance (Jahn et al., 2011; Pilate et al., 2012).

### BREEDING TOWARD CROP IMPROVEMENT

Although we emphasized the importance of genetic engineering for crop improvement, the importance of classical breeding should not be overlooked. Breeding is highly effective for polygenic traits and careful selection and genetic improvement toward specific properties over thousands of years have boosted the yield of the major food crops to such extent that most crops hardly resemble the wild varieties they were derived from Doebley (2004) and Smith and King (2000). A number of these crops have been further optimized for traits relevant to biomass production. For example, the current sugarcane yield almost doubled since the 1970s, from 45 tons/ha to approximately 80 tons/ha (Dal-Bianco et al., 2012). For other biomass crops (e.g., switchgrass and *Miscanthus*), intensive breeding programs have been initiated more recently. However, thanks to their relatively short life cycle and the integration of biotechnology in the breeding process, considerable progress toward biomass production was made over a short period of time (Stamm et al., 2012). This is in sharp contrast to trees grown for lignocellulosic biomass production. Although intensively used for thousands of years, trees were only recently cultivated and their long juvenile stage considerably slows down breeding programs. For example, the genetic improvement of poplar, considered worldwide as a promising lignocellulosic biomass crop, has only started in the late 18th century with the selection of inter-specific hybrids that appeared spontaneously (Flachowsky et al., 2009; Stanton et al., 2010). Modern breeding programs started much later, and with breeding cycles of 15–20 years, most advanced poplar breeding programs are in the fourth generation only. During recent years, considerable progress has been made in reducing the generation time of trees to accelerate breeding. Early flowering can now be induced by the application of plant hormones such as gibberellic acid or by transgenic techniques. For example, the overexpression of regulatory genes such as *LEAFY* (*LFY*) or *APETALA1* (*AP1*) results in flower induction and trees with a generation time of one year only (Pena and Seguin, 2001; Martin-Trillo and Martinez-Zapater, 2002).

The preferred breeding strategy is dependent on the physiological and hereditary characteristics of the crop. For example, some promising biomass crops are inter-specific hybrids and sterile (e.g., the allotriploid *Miscanthus* × *giganteus*), complicating breeding efforts. Here, chromosome doubling has been used to successfully restore fertility (Yu et al., 2009). Similarly, many potential energy crops are self-incompatible and obligate out-crossers, with large genetic diversity both within and among populations. This implies that successful breeding tools developed for inbred lines (cfr. maize) are not applicable for these crops. In addition, many domestication mutations are recessive defective alleles which generally explain a larger portion of the phenotypic variation than functional alleles, making it worthwhile to identify them. In breeding programs with self-incompatible plants, these rare recessive alleles will only manifest themselves in back-cross progeny that typically shows inbreeding depression. New technologies based on next generation sequencing, such as breeding with rare defective alleles (BRDA), in which large populations are screened for specific deleterious mutations in the gene of interest, can bring outcome (Marroni et al., 2011; Vanholme et al., 2013). The increase in sequencing capacity also allows genome-wide association studies (GWAS) to speed up breeding progress (Morris et al., 2013). These approaches can nowadays be extended to larger populations than currently used for breeding, even allowing to exploit the large genetic variation typically present in wild germplasm (Vanholme et al., 2013).

### CELL WALL IMPROVEMENT

**FIGURE 2:**
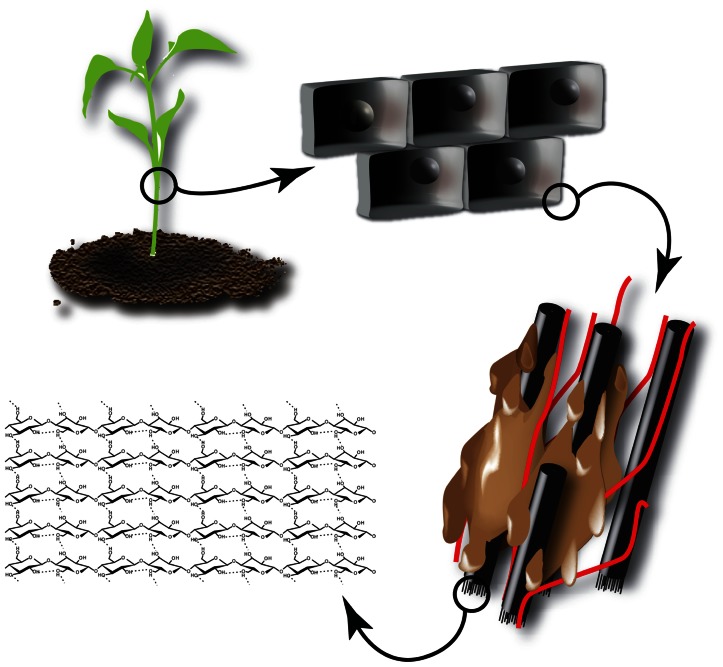
**Structure of cellulose within the plant cell wall**. The plant cell is surrounded by a recalcitrant cell wall composed of cellulose (β-1,4-coupled glucose monomers) aggregated into microfibrils (black). Microfibrils are crosslinked by hemicelluloses (red) and the resulting polysaccharide network is embedded in lignin (brown).

The main component of plant biomass used as second generation feedstock is the plant cell wall, which is composed of different polymers. In the case of the primary cell wall, these are mainly cellulose, hemicellulose, and pectin polysaccharides (Carpita and Gibeaut, 1993). Depending on the cell type and fate, a secondary cell wall containing cellulose, hemicelluloses, and lignin is deposited (**Figure 2**). The complex network of different polymers provides the strength that is needed to withstand mechanical stress and forms an important barrier to protect the cell against pathogens. This inherent recalcitrance makes it difficult to dismantle the cell wall and recover the desired building blocks for further processing. Here, green biotechnology holds an enormous potential to design cell walls for easier processing (Chen and Dixon, 2007; Gomez et al., 2008; Abramson et al., 2010; Carpita, 2012).

Increasing cellulose synthesis is an obvious strategy to improve the value of bioenergy crops. However, as the molecular mechanisms behind cellulose synthesis and deposition are still not fully understood, this approach is challenging (Guerriero et al., 2010). Nevertheless, a molecular pathway regulating cellulose biosynthesis should exist, since plants can change their cellulose content under specific conditions. For example, under mechanical stress trees produce tension wood which is characterized by a cell wall layer (the G-layer) that is composed almost exclusively of cellulose. As tension wood releases approximately 2.5× more glucose compared to control wood in saccharification experiments (Brereton et al., 2011), activating these mechanisms in trees is considered a Holy Grail to boost the bio-based economy. However, despite being intensively studied (Andersson-Gunneras et al., 2006; Kaku et al., 2009; Foston et al., 2011), the molecular mechanisms regulating tension wood formation are still not known and little progress is being made in this field.

Tension wood also contains considerably less lignin which will contribute to its enhanced saccharification efficiency. Indeed, lignin is a complex, recalcitrant and insoluble polymer that is considered the main (although not the only) limiting factor in the conversion of lignocellulose toward fermentable sugars (Chen and Dixon, 2007; Vanholme et al., 2008; Koo et al., 2012; Van Acker et al., 2013). In trees, lignin makes up 15–36% of the dry weight of wood (Zobel and van Buijtenen, 1989) and reducing this fraction is an attractive approach to improve biomass properties (Vanholme et al., 2010b). The percentage in lignin reduction required to obtain positive effects on saccharification efficiency largely depends on the plant species, as well as the composition and microarchitecture of the cell wall (Chen and Dixon, 2007; Centi et al., 2011). Because lignin is an important component of the secondary cell wall, reducing its abundance is often associated with a growth penalty (Van Acker et al., 2013). Whereas these growth defects were previously assigned to an overall reduction in mechanical strength of the cell wall, recent evidence links them at least in part to an accumulation of salicylic acid, a plant growth regulator directly derived from the phenylpropanoid pathway (Gallego-Giraldo et al., 2011).

Rather than reducing the lignin content, engineering the structure of lignin could be another elegant way to reduce cell wall recalcitrance (Grabber et al., 2012; Vanholme et al., 2012). The lignin polymer is composed of a few major aromatic building blocks (coniferyl alcohol, sinapyl alcohol, and minor amounts of *p*-coumaryl alcohol; Boerjan et al., 2003). Once coupled, these buildings blocks are called guaiacyl (G), syringyl (S), and *p*-hydroxyphenyl (H) units, respectively. The coupling generates a variety of chemical bonds, each having its own susceptibility to chemical degradation during downstream processing of the biomass. Altering the ratio of the major units of the lignin polymer can affect the ease of lignin degradation through chemical hydrolysis and can significantly improve saccharification and pulping (Pilate et al., 2002; Bose et al., 2009; Sannigrahi et al., 2010). However, as the shift in S/G ratio can come with a change in overall lignin content, conclusions should be interpreted with care (Vanholme et al., 2008; Stewart et al., 2009). Currently, both increases (Li et al., 2010) and decreases (Fu et al., 2011) in S/G ratio have been linked with improved biomass processing, whereas still other studies found no correlation between the lignin composition and saccharification yield (Chen and Dixon, 2007; Mansfield et al., 2012). Thanks to our current insight into the lignin biosynthetic pathway, plants can be steered to engineer entirely novel lignin polymers with vastly different properties (Baucher et al., 1996; Pilate et al., 2002; Leplé et al., 2007; Ralph et al., 2008; Vanholme et al., 2010a). In addition to the use of plant genes, a plethora of genes derived from other taxa might be used to generate novel building blocks and engineer easily degradable lignin polymers (Vanholme et al., 2012). For example, Eudes et al. (2012) have used a bacterial hydroxycinnamoyl-CoA hydratase-lyase (HCHL) to convert precursors of monolignol building blocks in non-conventional monomers, such as hydroxybenzaldehydes and hydroxybenzoate. Overexpressing this gene in *Arabidopsis* resulted in plants with shorter lignin polymers and an increased saccharification yield.

Although the focus so far has been on the reduction of lignin, biomass with high lignin content could also be interesting for applications other than saccharification. Lignin has a high calorific value, releasing more energy compared to polysaccharides when burned. Consequently, some biomass types with high lignin concentration (e.g., endocarp) have an energy content comparable to that of charcoal (based on the heating value), and higher than that of classical energy crops (Mendu et al., 2012). In addition, lignin could be a renewable resource of valuable building blocks for the chemical industry, and replace fossil fuel (petroleum)-based polymers. Despite its great potential in this field, the heterogeneity of the lignin polymer makes it difficult to process (Chung and Washburn, 2012).

**FIGURE 3:**
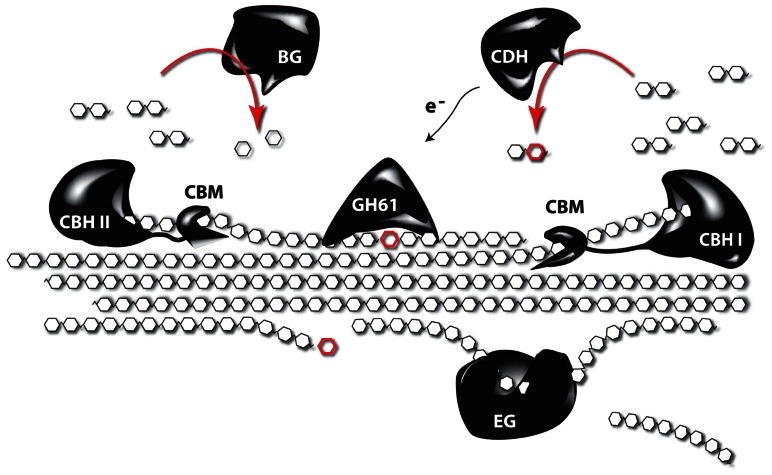
**The synergistic function of different enzymes to degrade crystalline cellulose**. Complete depolymerization of celluloses is obtained by the synergistic action of several enzymes. Endoglucanases (EG) cleave internal bonds at amorphous sites and create new chain ends. Exocellulases or cellobiohydrolases (CBH) cleave two units from the ends of the cellulose polymer, releasing disaccharides (cellobiose). There are two types of CBH: one working from the reducing end (CBH I), and another working from the non-reducing end of cellulose (CBH II). β-glucosidases (BG) hydrolyze the oligosaccharide products into individual monosaccharides. In turn, cellobiose dehydrogenases (CDH) use an acceptor molecule to oxidize cellobiose by a radical reaction. The released electrons can be used by polysaccharide monooxygenases, such as those from glycosyl hydrolase 61 (GH61), to depolymerize crystalline cellulose through reductive elimination. Most of the enzymes are coupled to carbohydrate-binding modules (CBMs), which have no catalytic activity, but secure the binding of the catalytic domain to the polysaccharide (based on Horn et al., 2012).

Despite the many studies linking lignin amount to cellulose accessibility, there is growing evidence that factors beyond lignin content influence cell wall recalcitrance (Studer et al., 2011; Brereton et al., 2012; Ray et al., 2012; Van Acker et al., 2013), bringing factors such as the lignocellulosic architecture or hemicellulose content and composition into the picture. Indeed, enzymatic hydrolysis of hemicelluloses is an efficient way to improve the saccharification potential, but the overall complexity of the hemicellulose structures asks for a cocktail of enzymes for their degradation into oligo- and monosaccharides. In addition, a substantial fraction of hemicellulose-derived sugars are pentoses (xylose and arabinose) and these sugars cannot be fermented by the yeast strains used to produced ethanol on an industrial scale (Chandel et al., 2010, 2012). Tuning the hemicellulose content or composition of the biomass is a promising strategy to circumvent these problems. One way to do this is to degrade part of the cell wall by the expression of genes encoding cell wall-degrading enzymes (CWDEs) during plant growth (Obro et al., 2011). Although CWDEs targeting the major polysaccharides of the cell wall have already been expressed in plants, in most studies the effect on saccharification yield has not been investigated or a profound cell wall characterization of these plants is lacking. In addition, the focus has mainly been on the expression of single genes, whereas it is known that CWDEs work synergistically, and complex enzyme cocktails are needed to degrade the cell wall (**Figure 3**). Obviously, a complete degradation of the cell wall during development is not the aim of such approaches, however, it would be interesting to combine enzymes, since such combination could have additive effects (Zhang et al., 2011). Such approaches, where plant cell walls are modified, will always ask for a subtle balance between the modification level to improve downstream processing and the avoidance of a negative impact on plant physiology. Not only will the cell wall be weakened by the partial degradation, also cell wall fragments will be released in the apoplast. These fragments can act as elicitors that activate stress response pathways in the plant (Ridley et al., 2001). The penetrance of such response can depend on the environmental conditions (Taylor et al., 2008), underscoring the importance of field trials to test the performance of these novel biomass crops under more adverse conditions, such as greater variability in light, wind, and temperature. To avoid possible adverse effects on plant growth and development, enzymes of thermophilic organisms are being used. The underlying idea is to produce CWDEs that are inactive during growth and development but can be activated upon harvest by submerging the biomass in hot water. This strategy was further optimized by inserting the code for a thermostable self-splicing bacterial intein in the coding sequence of the enzymes (Shen et al., 2012). Because the enzymes are produced by the plant, it limits the requirement for complex and expensive enzyme cocktails to efficiently saccharify the biomass, thus significantly reducing the processing costs (Sticklen, 2006; Oraby et al., 2007; Borkhardt et al., 2010). As the targeted substrates will be most likely embedded in lignin by the time the enzymes are activated, improved processing is to be expected in plants engineered to make less lignin. Other strategies target the cell wall microarchitecture rather than the lignin polymer to increase cell wall porosity and improve saccharification yield. This can be obtained by the heterologous expression of genes coding for non-catalytic carbohydrate-binding modules (CBMs). These will destabilize the cell wall by intercalating between cell wall polysaccharides. Interesting, besides modifying the architecture of the plant cell, the accumulation of CBM in the cell wall generally has a positive effect on the plant yield (Shoseyov et al., 2006). Alternatively the incorporation of non-crystalline soluble polysaccharides (e.g., hyaluronan) in the cell wall might result in plants with normal structural integrity, but as these polysaccharides are soluble, they are expected to be easily removed from the cell wall during pretreatment steps, leaving behind a more porous cell wall (Abramson et al., 2010).

## THE WHITE BIOTECHNOLOGY PILLAR: FROM BIOMASS TO PRODUCT

### BIOMASS PRETREATMENT

The conversion of fermentable sugars into chemicals, materials, or fuels is achieved with microorganisms and their enzymes, which are the workhorses of white biotechnology. Although this discipline has a very rich history, it is only recently that lignocellulosic biomass is used as a source for sugars. Due to differences in its composition and complexity compared to classical fermentation feedstocks (e.g., sucrose or starch), this shift came with new challenges (Chandel et al., 2012). Although the biomass itself can be optimized for downstream processing, a physicochemical pretreatment of biomass is essential to open up the substrate’s structure and lower its recalcitrance. The subsequent biological conversion then typically consists of two steps, i.e., enzymatic hydrolysis followed by microbial fermentation – although both can also be performed simultaneously.

Despite numerous efforts, the establishment of an ideal pretreatment procedure has not yet been achieved. In contrast, the most optimal protocol has been found to depend heavily on the type of biomass that needs to be processed. This implicates that a one-size-fits-all solution is unlikely to exist (Hendriks and Zeeman, 2009). Furthermore, the pretreatment procedure also has a significant impact on the subsequent hydrolysis and fermentation steps, and should thus not be evaluated independently. Treatment with strong acids, for example, is well known to generate side products (e.g., furfural, 5-hydoxymethylfurfural, levulinic acid, and formic acid) derived from hemicellulose pentoses that have an inhibitory effect on microorganisms used during fermentation. Nevertheless, some criteria have been proposed that can be used to evaluate the usefulness of a pretreatment procedure (da Costa Sousa et al., 2009). These not only include technological factors, but also environmental and economic parameters. In contrast to the other steps in the conversion process, pretreatment is still relatively expensive and can account for up to 15–20% of the total capital investment.

Pretreatments involving high temperatures (>160°C) have been successfully applied to a wide variety of biomass types. The main goal is to solubilize hemicelluloses (and to a lesser extent lignin), and thus to make cellulose more accessible. Steam explosion probably is the most efficient example but carries the risk of inhibitor formation, especially under acidic conditions (Ballesteros et al., 2006). Although acids can be used in concentrated solutions, they are often diluted (e.g., ~1% sulfuric acid) to limit hydrolysis and secondary reactions of hemicelluloses (Chen et al., 2012a). However, acid hydrolysis of hemicelluloses can eliminate the need for additional enzymes such as xylanases, which significantly lowers the cost of the overall process. In any case, the resulting biomass needs to be neutralized by extensive washing before it can be fermented by microbes. Alkaline pretreatments, in contrast, mainly act on the ester linkages between lignin and carbohydrates, which allows the aromatic polymer to be extracted from the biomass. A famous example is ammonia fiber expansion (AFEX), where concentrated ammonia (~1 kg/kg dry biomass) is applied at relatively low temperatures (~100°C) in a high-pressure reactor (Balan et al., 2009). The volatile catalyst can afterward easily be recycled, leaving the biomass ready for hydrolysis and fermentation without prior detoxification. Alternatively, fractionation of biomass components can also be achieved with specific solvents. In the organosolv process, for example, aqueous alcohol in the presence of dilute acid (~60% ethanol, 1% H_2_SO_4_) is used to extract lignin and hemicelluloses, respectively (Zhao et al., 2009). Although this technology is successfully applied in the pulp industry, it is rather expensive for biofuel production. Finally, oxidizing agents such as hydrogen peroxide or peracetic acid can also be used to remove hemicelluloses and lignin from biomass. However, these chemicals are rather unspecific and can result in a significant reduction in sugar yields, as well as formation of inhibitors derived from lignin.

### ENZYMATIC HYDROLYSIS: LOWERING THE ENZYME COST AND OPTIMIZING THE ENZYME COCKTAIL

Several microorganisms are able to grow on lignocellulosic biomass and can thus be used as source of hydrolytic enzymes (Gilbert, 2010). Many cellulolytic bacteria produce a multi-enzyme complex called cellulosome, in which various carbohydrate-degrading activities are linked to a central scaffold that is physically attached to the bacterial cell wall (Fontes and Gilbert, 2010). Fungi, in contrast, secrete a mixture of soluble enzymes into the extracellular medium. As the enzymes are much easier to recover in that case, fungal cellulases have become the preferred constituents of commercial preparations (van den Brink and de Vries, 2011). These basically consist of three enzyme classes: endoglucanase, exoglucanase or cellobiohydrolase (CBH) and β-glucosidase (**Figure 3**). For the degradation of hemicellulose, however, a much broader range of specificities is required, including xylanases, α-L-arabinofuranosidases, mannanases, α-galactosidases, and multiple esterases (Shallom and Shoham, 2003). The most famous source of cellulolytic enzymes undoubtedly is the saprophytic mesophilic fungus *Trichoderma reesei* (now reclassified as *Hypocrea jecorina*). The enzyme cocktail that it produces consists of two exo- and at least five endoglucanases that act synergistically to degrade cellulose. The former are by far the most abundant components, with CBH I even making up about half of the secreted protein. Although the secretome of *T. reesei* was found to contain many additional catalytic and auxiliary proteins (Foreman et al., 2003), the sequencing of its genome revealed that it encodes fewer (hemi)cellulases than any other fungus able to decompose plant biomass (Martinez et al., 2008). Despite the fact that *T. reesei* is well established as industrial workhorse, efforts are still being directed toward the identification of new cellulolytic organisms with improved properties (Gardner et al., 2012; Bhalla et al., 2013).

Cellulases are relatively costly enzymes, which has long been one of the major hurdles for the commercialization of cellulosic ethanol. During the past decade, however, their cost has decreased more than 20-fold to about US$0.12/gallon ethanol, mainly thanks to efforts from Novozymes and Dupont Industrial Biosciences (formerly Genencor) in subcontract with the US Department of Energy (Merino and Cherry, 2007). This impressive result has been achieved by a combination of increased enzyme performance and improved enzyme production. Although the wild-type *T. reesei* strain QM6a is already able to secrete high amounts of protein, numerous mutants that perform even better have been isolated (Peterson and Nevalainen, 2012). At Rutgers University, for example, random mutagenesis has famously been applied to create strain RUT-C30 that secretes up to 20 g/L protein. Furthermore, this strain is less sensitive to catabolite repression by glucose, which enabled its use as inducer (in combination with cellulose). Although sophorose is the most efficient inducer of cellulases, its excessive price prohibits its application in commercial processes. It can, however, be produced *in situ* from glucose by the transglycosylation activity of cellulases (England et al., 2010), but this process is not very efficient. Therefore lactose has long been used as alternative but the ability to switch to glucose decreased the cost of induction even further. Finally, optimization of the fermentation process itself has also resulted in major gains in the cost-benefit balance (Singhania et al., 2007).

Considerable progress has also been made in the optimization of the individual components of the enzyme mixture. Cellulases are rather slow enzymes, and increasing their specific activity has, therefore, long been on the wish list of protein engineers, but the results have been moderate at best (Wilson, 2009). One of the main challenges is that enzyme improvements as measured with one (artificial) substrate do not necessarily translate into improved performance in industrial settings. In addition, the moderate thermostability of the mesophilic *T. reesei* enzymes has been addressed by random and rational mutagenesis. In that respect, CBH I has been one of the main targets since it is the most abundant, but also the least stable cellulase (Tm ~61°C). However, its melting temperature could be increased with almost 15°C, making it at least as stable as the other enzymes (Lantz et al., 2010). In parallel, improved homologs have also been identified in nature and used to complement the enzyme cocktail of *T. reesei*. The cloning of a glucose-tolerant β-glucosidase, for example, has largely solved the problem of product inhibition during hydrolysis (Mathew et al., 2008). Somewhat similar to the situation with pretreatment protocols, the goal of composing a perfect enzyme mixture for the hydrolysis of lignocellulosic biomass has been abandoned for the development of dedicated cocktails tailored to a specific type of biomass and pretreatment technology (Levine et al., 2011). For example, the original cellulase mixture from Dupont Industrial Biosciences has evolved into Accellerase DUET and TRIO, which are supplemented with auxiliary enzymes (β-glucosidase and hemicellulase). It is clearly stated in the application notes of these products that “when pretreatments result in a feedstock that would benefit from additional hemicellulose degradation, one should test whether there is synergy between the different enzyme samples.”

Next to the hydrolytic enzymes, the existence of auxiliary proteins that stimulate the activity of cellulases has been debated ever since the early days of biomass research (Reese et al., 1950). Their true identity was, however, only revealed a few years ago. Indeed, a protein was identified that displays significant homology to plant expansins and disrupts the crystalline structure of cellulose without apparent hydrolytic activity (Saloheimo et al., 2002). This so-called swollenin was, however, later found to slowly hydrolyze hemicellulose components, which could be a possible explanation for the observed effect (Yao et al., 2008). Clearly, more research is needed to completely unravel its mode of action and to evaluate its contribution to the efficient degradation of biomass. In parallel, members of the glycosyl hydrolase family 61 (GH61) were recently shown to degrade cellulose by means of an oxidative instead of a hydrolytic mechanism (Westereng et al., 2011; **Figure 3**). These proteins were long described as cellulase-enhancing factors, but can now be designated as polysaccharide monooxygenases. They do not have the typical substrate-binding cleft observed in canonical cellulases, but seem to bind linear carbohydrate chains along their flat surface where the active site is located (Quinlan et al., 2011). Their ability to depolymerize crystalline substrates significantly facilitates the activity of cellulases, meaning that lower enzyme loads are needed and perhaps even less severe pretreatment conditions (Harris et al., 2010). Furthermore, their physiological redox-partner seems to be cellobiose dehydrogenase, which acts on the product of cellulose hydrolysis and thus provides positive feedback to the oxidation–hydrolysis reaction sequence (Langston et al., 2011). These discoveries represent major breakthroughs in cellulose research and are expected to make the conversion of biomass considerably more cost-effective.

### CONVERSION OF BIOMASS TO PRODUCTS – TAILOR MADE MICROORGANISMS

Simple organic compounds derived from the primary metabolism (i.e., ethanol, butanol, or acetone) were the first set of chemicals to be targeted for microbial overproduction at an industrial scale (Jones and Woods, 1986). However, the ambition of many countries to become economically independent from fossil fuels prompted the production of an increasing number of fine and bulk chemicals, many of which were never produced before by these microbial hosts. Emerging fields, such as metabolic engineering, protein engineering, and synthetic biology, consolidate this transition and the specific microorganisms used in fermentation reactions are increasingly engineered to efficiently produce the desired compound. These “designer bugs” can be considered as real cell factories that are used to produce complex metabolites or their direct precursors with a myriad of applications in, among others, antibiotics (cephalexin), pharmaceuticals (artemisinin, taxol, resveratrol, and palifosfamide), food additives (vanillin, valencene, succinic acid, citric acid, and amino acids), cosmetics (farnesene), surfactants (rhamnolipids and sophorolipids), and chemicals (D-lactic acid and isobutanol) (Park et al., 2007; Lee et al., 2009; Tsuruta et al., 2009; Ajikumar et al., 2010; Papini et al., 2010; Adsul et al., 2011; Van Bogaert et al., 2011).

In contrast to the intensive efforts to obtain novel metabolites, tuning the organisms has not been considered the main priority in optimizing the production of bulk chemicals. Many of these classical fermentation processes have a rich history, and the specific hosts have been carefully selected over decades or even centuries (Solomon et al., 2007). Consequently, most are robust and outperform other microorganisms under the variable processing conditions used during fermentation. For example, *Saccharomyces cerevisiae* readily produces ethanol from glucose, mannose, or fructose and additional engineering of this organism was not considered crucial for the production of first generation biofuel (van Maris et al., 2006; Solomon et al., 2007). This philosophy changed considerably with the shift to second generation feedstocks and the task to develop an economy that has to compete with the fossil-based industry. This challenge urged to improve the fermentation process at all levels, including the microbial host. One of the important shortcomings of *S. cerevisiae*, limiting its use in processing lignocellulosic biomass, is the lack of a metabolic mechanism to ferment pentose sugars (constituting 10–20% of the total dry weight in woody biomass; van Maris et al., 2006). Although the xylose content of the biomass can be reduced by modifying the plant cell wall or by introducing specific pretreatment steps in the processing pipeline(as discussed before), it would be a tremendous benefit to use an organism that could simultaneously convert both sugar types to ethanol (Solomon et al., 2007). Pentose fermenting organisms exist, and environmental samples have been successfully explored for new species or strains with promising and useful properties (Taguchi et al., 1994; Suh et al., 2003, 2005; Epting et al., 2005; Kuhad et al., 2011). However, so far these isolated microorganisms suffer from some physiological drawbacks such as low product tolerance, long fermentation periods, or the production of considerable amounts of byproducts, making them currently commercially unviable (Kuhad et al., 2011). Although these organisms can be further optimized, modifying a microorganism into an industrially robust host is challenging. Nevertheless, the study of these organisms can help in the genetic engineering of *S. cerevisiae* to create a xylose fermenting yeast strain. An attractive and successful approach is the introduction of a functionally active bacterial xylose isomerase in the yeast to convert xylose to xylulose that is introduced via the pentose phosphate pathway in the yeast’s primary metabolism (Brat et al., 2009). Alternatively, xylose can be converted to xylulose by a two-steps reaction using xylose reductase (XR) and xylitol dehydrogenase (XDH)*.* Overexpressing both genes resulted in a yeast strain with an efficient conversion of pentoses as sole carbon source (Jeffries, 2006). However, promising laboratory strains are currently not sufficiently robust to perform equally well under industrial conditions using complex lignocellulosic-derived sugar mixtures as substrate and further improvement is desirable to reach economically feasible performance.

Besides broadening the substrate specificity, increasing the efficiency of ethanol production is another strategy to reduce fermentation costs and bring bio-ethanol into a competitive position with fossil fuels. This could be achieved by channeling glucose through the Entner–Doudoroff (ED) pathway toward ethanol. This pathway is used by only few organisms among which the gram negative ethanol producing bacteria *Zymomonas mobilis *(Seo et al., 2005) and results in an overall higher ethanol yield (~10% more ethanol per fermentable glucose) compared with the more common, but less efficient Embden–Meyerhof (EM) pathway. Replacing the complete EM pathway in yeast by the ED pathway would be a logical strategy, but swapping metabolic pathways is challenging and although patented, this approach is so far not further explored (Lancashire et al., 1998; de Kok et al., 2012). Optimizing *Zymomonas* for industrial ethanol production is in this case a more feasible approach, especially since this organisms also has a high ethanol tolerance (up to 14%; for *S. cerevisiae* this is ~11% at 30°C). The thermophilic organism *Clostridium thermocellum* is studied for a similar reason. Although its ethanol tolerance is somewhat lower compared to *Zymomonas* (~12%), this organism secretes considerable amounts of CWDEs, offering the prospect to produce ethanol directly from biomass by combining hydrolysis and ethanologenesis. Moreover, as *C. thermocellum* is thermophilic, fermentation can be performed at temperatures where the risk of contamination is almost inexistent (Chang and Yao, 2011) with the additional benefit that the elevated temperature decreases the viscosity, increases reaction rates and facilitates ethanol recovery (Taylor et al., 2009; Chandel et al., 2013). Without doubt, both *Clostridium* and *Zymomonas* are promising organisms with interesting properties for the production of bioethanol and they were predicted to outcompete the industrial yeast strains used today for the commercial production of bioethanol (Lin and Tanaka, 2006). However, despite being intensively studied and modified, the organisms still lack the robustness of the industrial yeast strains and the produced ethanol concentrations are at maximum when utilizing only glucose as carbon source, making them currently less suitable to support the bio-based economy running on lignocellulose. Nevertheless, the available genetic engineering toolbox combined with upcoming state of the art technologies (Callura et al., 2012) will significantly improve the properties of these, as well as of other microorganisms.

### ANAEROBIC DIGESTION TO RECOVER ENERGY

Following the conversion of biomass to added value products by fermentation, the organic waste stream generated during this process can be reduced and partly converted to energy (i.e., gas) by anaerobic digestion (Kleerebezem and van Loosdrecht, 2007; Tilche and Galatola, 2008). The biogas produced by this process is a mixture of methane and carbon dioxide with typically a low amount of hydrogen sulfide and trace compounds depending on the nature of the feedstock (Vandevivere et al., 1998; Tsavkelova and Netrusov, 2012). It is generally observed that the conversion of 1 kg dry biomass delivers about 0.5 m^3^ of biogas at a 60/40 methane/carbon dioxide ratio (Vandevivere et al., 1998). This gas can either be purified, using approaches such as pressure swing absorption, to achieve an upgraded gas equivalent to natural gas or it can be combusted on site in a “combined heat and power” (CHP) approach that delivers about 1 kWh electric power per kg dry biomass converted, and 3 kWh heat (Angenent et al., 2004). The latter can be used to heat up fermenters or to evaporate water. This may be critical to the operation of a biorefinery producing diverse chemicals, as typically fermentation water needs to be added to the incoming biomass. To facilitate the final processing of residual biomass, as e.g., described in the next section, dewatering is essential.

The decomposition process from biomass toward biogas can be divided into four sequential phases (Angenent et al., 2004). Hydrolysis converts the organic polymers into oligo- and monomers (mainly amino acids, sugars, and fatty acids). These are converted by acidogenesis into organic acids and alcohols, which are subsequently transformed into acetic acid, carbon dioxide, and hydrogen by acetogenesis. The final step, methanogenesis, leads to the production of methane and carbon dioxide from acetate (acetoclastic) or hydrogen (hydrogenotrophic). Achieving the effective degradation of complex organic substrates toward single carbon products over the different phases is not feasible with a pure microbial culture. Instead the conversion is performed by a complex but undefined microbial community comprising both Bacteria (*Clostridia, Bacilli*, etc.) and Archaea [type strains *Methanosaeta* or *Methanosarcina *(Riviere et al., 2009)]. Typically, Eubacteria are implicated in hydrolysis, acidogenesis, and acetogenesis, whereas the last step of the process (methanogenesis) is performed by Archaeal methanogens converting acetate, hydrogen, methanol, or single carbon amines to methane. The presence of all trophic levels is required within the anaerobic digester, which necessitates controlled conditions in terms of pH, temperature, and possible toxic byproducts. For example, Archaea operate within a narrow pH range (~6.5–8.5) below which free acid inhibition or sulfide inhibition occurs, whereas at higher pH ammonia toxicity has been observed (Angelidaki and Ahring, 1993). Acidogenic bacteria are more active at a somewhat lower pH range (5.5–6.0; Chyi and Dague, 1994). Crucial for the efficiency of the anaerobic digestion process is setting appropriate parameters, that balance all conversions running within the anaerobic digestion tank. The multitude of interactions between the different microorganisms of these complex and undefined cultures makes it impossible to model the individual conversions, hence the optimization of this process has in the past mainly been based on a trial and error approach (Lubken et al., 2010). Even the study of individual biochemical processes running within the reactor is a complicated task. Most species isolated from a bioreactor cannot be grown under optimized laboratory conditions in monoculture, and, if isolated, studies on pure strains are mostly inappropriate as they give a distorted view of their role within a microbial community. In recent times the emergence of high throughput sequencing approaches and particularly combined metagenomics/metatranscriptomics has allowed new insights into reactor operation and microbial ecology (Simon and Daniel, 2011). Metagenomics on biogas producing microbial communities has given a glimpse inside the anaerobic digestion reactor and revealed a community composed of over 1000 different bacterial species including numerous, so far unidentified microbes (Wirth et al., 2012). Besides the identification of the species within the reactor, this technique allows studying the behavior and shift of bacterial populations within communities over time, depending on growth conditions and substrate availability (Hollister et al., 2010, 2011). The next step in the study of this microbial community is to truly steer it toward optimal performance.

In the slipstream of anaerobic digestion, the fermentation of side streams toward carboxylates is an emerging field of research. The so-called carboxylate platform, enabled by this approach, promises the production of a plethora of fuels and chemicals from biorefinery side streams, thereby altering the economics of the overall biorefinery, as well as delivering novel products (Agler et al., 2011), such as longer chain carboxylates and their derivatives. In a similar context, fermentation products can be upgraded toward more attractive products using electricity as source of reducing/oxidizing power, thereby integrating biomass feedstocks with renewable energy within one concept (Rabaey and Rozendal, 2010; Rabaey et al., 2011). Similar electricity using technology can further be deployed on the biorefinery side streams to recover other products, such as sodium hydroxide, which typically lead to salt accumulation in closed or limited water cycles (Rabaey et al., 2010).

## THE THERMOCHEMICAL PILLAR: CONVERSION OF RESIDUAL BIOMASS STREAMS

### CONVERSION OF BIOMASS WASTE TO BIO-OIL AND BIOCHAR

To close the carbon cycle of the sustainable economy, the organic matter containing residue streams from biorefinery processes and anaerobic digestion are thermochemically converted into bio-oil and biochar (i.e., by means of pyrolysis). This process can be integrated in a production pipeline to maximize energy recovery in combination with the production of specific added value products. The bio-oil has different properties compared to petroleum, but can be combusted to generate electricity. The produced biochar is brought back into the soil, ensuring sequestration of carbon and nutrients, thereby closing the nutrient cycle while improving sustainable soil fertility and crop productivity.

Distinction can be made between fast pyrolysis and slow pyrolysis (or carbonization; Brown, 2009; Duku et al., 2011). In fast pyrolysis, reaction conditions are selected to obtain a maximum yield of condensable vapors (forming the bio-oil). These conditions are moderate temperatures (400–600°C), rapid heating rates (>100°C/min) combined with short residence times of the biomass particles (0.5–2 s), small particle size to support high heating rates (typically less than a few millimeters) and rapid cooling or quenching of the pyrolysis vapors into bio-oil (Czernik and Bridgwater, 2004; Balat et al., 2009; Bridgwater, 2012). These fast pyrolysis conditions result in bio-oil yields up to 70–80% with limited levels of char yield (around 12%). To promote the maximum yield of the solid fraction (char), slow pyrolysis employs lower heating rates and vapor residence times higher than 10 s at low to medium temperatures (450–650°C; Demirbas, 2004). The longer vapor residence times ensure secondary cracking reactions in which the vapor phase constituents are further broken down into additional (secondary) char and non-condensable gases (Di Blasi, 2008). Under these conditions, char yields up to 35% could be attained (Bridgwater, 2012).

### ENVIRONMENTAL AND AGRONOMIC BENEFITS

The major benefit of the thermal treatment that biomass undergoes during biochar production, is that the biomass constituents are converted into a recalcitrant, carbon-rich material that has been demonstrated to be very stable once stored in the soil, with a half-life ranging between several hundreds to over 1000 years (Kuzyakov et al., 2009; Lehmann and Joseph, 2009; Zimmerman, 2010). The stability of biochar is further corroborated with the discovery of the Amazonian dark earths, better known as *terra preta*, which are fertile soils, still rich in organic carbon due to the presence of charcoal, which is believed to have been deliberately and systematically deposited by pre-Columbian farmers, some 800–5000 years ago (Smith, 1980; Glaser et al., 2002; Lehmann et al., 2003; Glaser, 2007; Lehmann, 2007; Barrow, 2012). Because of its resistance to biological decay, biochar has the potential to store carbon that has been removed as carbon dioxide from the atmosphere during photosynthesis and prevents the rapid release of carbon dioxide that would originate from biological decay if the biomass would be kept untreated (Woolf et al., 2010). Biochar production is therefore one of the few technologies that can actively remove existing carbon dioxide from the atmosphere, as opposed to merely capturing it at the point of emission or reducing its emission. According to Lehmann (2007), one hectare of arable land can store up to 250 tons of carbon and if biochar utilization would be adopted globally, it has been estimated that up to 5.5–9.5 × 10^9^ ton carbon could be sequestered worldwide on a yearly basis, potentially off-setting all current anthropogenic fossil fuel emissions of ca. 9 × 10^9^ ton carbon a year (Lehmann et al., 2006; Mathew et al., 2008; Lehmann and Joseph, 2009).

Although the potential of biochar to sequester carbon is a clear benefit, another major reason why biochar is recently gaining interest globally is the potential increase in soil productivity resulting from the changes in chemical and physical soil properties brought upon by biochar as a soil amendment (Chan et al., 2007; Laird et al., 2009; Novak et al., 2009; Major et al., 2010; Galinato et al., 2011). The meta-analysis by Jeffery et al. (2011) – covering 16 of these studies relating to biochar amended soil productivity – demonstrated that the average crop yield (or productivity) increased by 10%. Considering that crop yield is the result of a complex relationship between climate, soil type, crop type, and the physicochemical characteristics of biochar, it is not possible to draw a general conclusion regarding crop productivity yield increase in biochar amended soils. It is equally important to stress that biochar is not a singular product, but a term given to cover a wide range of carbonaceous products obtained from different pyrolysis processes and from different biomass feedstock types (Spokas et al., 2012). Furthermore, up to now, most studies have been carried out on weathered soils in tropical regions, whereas data on crop productivity on younger soils in more temperate climate regions are still lacking (Galinato et al., 2011).

The mechanisms by which biochar increases soil fertility and crop productivity are not completely understood. Research has already demonstrated how biochar, through its unique physicochemical properties obtained during the biomass pyrolysis process, interacts with soil and soil microorganisms (Sohi et al., 2010; Barrow, 2012). Biochar is characterized as a highly porous structure having a large specific surface area, which can amount up to around 400 m^2^/g, depending on the biomass feedstock used and the pyrolysis conditions (Downie et al., 2009; Van Zwieten et al., 2009; Bruun et al., 2012; Chen et al., 2012b; Ronsse et al., 2013; Song and Guo, 2012). This highly porous nature not only results from the microstructure of the original biomass used to produce biochar (e.g., capillary structures such as xylem in woody biomass), but also from (physical) cracking due to mass loss and shrinking during the pyrolysis process. This highly porous nature of biochar induces several effects once added to the soil: the soil’s aeration and water holding capacity increase (Glaser et al., 2002; Jeffery et al., 2011; Karhu et al., 2011; Uzoma et al., 2011), while the pores provide a protective habitat against predatory soil fauna and desiccation, suitable for colonization by beneficial soil microorganisms (Warnock et al., 2007). Although biochar is mainly composed of highly recalcitrant carbon, i.e., chemically condensed polyaromatic ring structures (Amonette and Joseph, 2009), a fraction of labile, volatile organic compounds that are easily metabolized by soil microorganisms may be present. This is often observed by an initial and temporary increase in soil respiration (carbon dioxide production) after applying biochar, hence this effect is called “priming effect” (Novak et al., 2009; Jones et al., 2011; Zimmerman et al., 2011). However, if biochar has been produced through pyrolysis conditions that limit the occurrence of labile carbon compounds in the char, then this priming is less significant to non-observable at all (Cross and Sohi, 2011; Case et al., 2012).

The impact of biochar on the soil’s microbial ecology is also observed by a reduction in the non-carbon emissions by the soil. Reductions in nitrous oxide emissions ranging from 10 to 85% have been reported after amending the soil with biochar (Rondon et al., 2005; Yanai et al., 2007). Furthermore, biochar induces methane oxidation in soil, with one study reporting complete suppression of methane emissions from the soil (Rondon et al., 2005). Given the fact that the greenhouse warming potential of both nitrous oxide and methane is considerably higher than that of carbon dioxide (Forster et al., 2007), the potential reduction of these greenhouse gases could provide an additional climate change mitigation next to the carbon sequestration effect of biochar. In addition, through the pyrolysis process, biochar is characterized to have an alkaline pH and can therefore neutralize acidic soils (Lehmann et al., 2011). As biochar consists of a highly aromatic condensed structure, its surface – through exposure in the soil – oxidizes to form carboxylic groups (Cheng et al., 2008). In turn, these carboxylic groups provide an increase in the soil’s cation exchange capacity (Glaser et al., 2002). Consequently, the capacity of the soil to retain nutrients is increased and the losses due to leaching are decreased, which results in positive effects on crop yield (Duku et al., 2011).

With respect to nutrient management, biochar could prove to be essential to support sustainable biomass production for second generation biofuels (and biochemicals). As a large portion of the crop is being removed from the fields year after year to be valorized, there is a large risk in deteriorating the soil fertility by depleting organic carbon and nutrients. Counteracting these nutrient losses by mere supply of fertilizer, manure or compost is not sustainable in the long-term and could cause stream and groundwater contamination (Barrow, 2012). However, during pyrolysis for biochar production, the minerals are largely retained in the char, while almost 50% of the biomass nitrogen (Hossain et al., 2011; Liu et al., 2011) and nearly 100% of the biomass phosphorus (Bridle and Pritchard, 2004) end up in the char – although recovery rates are influenced by the pyrolysis conditions such as temperature (Liu et al., 2011). By pyrolyzing the residues streams from agriculture, biorefineries, and anaerobic digesters, the nutrients (including minerals, nitrogen, and phosphorus) are essentially recovered in biochar, thus closing the nutrient loop back to agricultural production, while the biochar-induced increase in soil cation exchange capacity ensures improved nutrient use efficiency. Due to its potential, considerable interest in biochar has been gained and in order to ensure its momentum and progress, some research needs are clearly identifiable: first, the interactions between biochar and soil need to be further unraveled, and the relationships between biochar’s physicochemical properties and soil fertility need to be established. However, the difficulty in this is to uncover the exact nature of biochar, given the variety of potential biomass feedstocks to be used and the different pyrolysis processes and process conditions. More research into biochar quality and the establishment of quality standards is needed (Barrow, 2012). With respect to carbon sequestration, assessing biochar stability in order to quantify its carbon potential is a key issue. And the final issue pertains to the economic feasibility of biochar production and under which production and implementation scenario biochar is economically profitable.

## PERSPECTIVES

Although the bio-based economy is still in its infancy, it is expected to grow exponentially as a consequence of the drive toward sustainable production processes, the strong price increases for fossil resources, and the need to reduce the emission of greenhouse gases. If further developed, the carbon-negative bio-based economy has the potential to play a key role in mitigating climate change. However, this will require an entire innovation chain that covers the integration of both fundamental and applied research. For this, the different disciplines should interact to design a flexible, cost-effective and waste-free processing pipeline from solar energy toward biomaterials and bioenergy. Initial efforts in the different disciplines resulted in a drop in processing costs already, but to further increase the efficiency and to provide economically viable alternatives to our current fossil fuel-based economy, the pathway should be tuned in an integrated way, as each modification in one of the steps will have repercussions on both up- and downstream processes.

## Conflict of Interest Statement

The authors declare that the research was conducted in the absence of any commercial or financial relationships that could be construed as a potential conflict of interest.
